# The Vaporization Enthalpy and Vapor Pressure of (±) *N*-Ethyl Amphetamine by Correlation Gas Chromatography

**DOI:** 10.3390/molecules26133809

**Published:** 2021-06-22

**Authors:** James S. Chickos

**Affiliations:** Department of Chemistry and Biochemistry, University of Missouri-St. Louis, 1 University Blvd, St. Louis, MO 63121, USA; jsc@umsl.edu Tel.: +1-314-5165377

**Keywords:** *N*-ethylamphetamine, S (+)-methamphetamine, vaporization enthalpy, vapor pressure, correlation gas chromatography

## Abstract

The vaporization enthalpy, and vapor pressure as a function of temperature of *N*-ethylamphetamine, a substance used in the 1950s as an appetite suppressant and more currently abused as a designer drug, is reported. Its physical properties are compared to those of S (+)-*N*-methamphetamine, a substance whose physiological properties it mimics. A vaporization enthalpy of (62.4 ± 4.4) kJ·mol^−1^ and vapor pressure of (19 ± 11) Pa at *T* = 298.15 K has been evaluated by correlation gas chromatography. Results are compared to estimated values and to the limited amount of experimental property data available.

## 1. Introduction

*N*-Ethyl amphetamine, also known as etilamfetamine, marketed as the hydrochloride salt under the trade name, Apetinil^TM^, was sold as an appetite suppressant in the 1950s [1]. It has been abused as a recreational stimulant, a “designer drug” alternative to amphetamine and possibly (+) S-methamphetamine [2]. Unlike R (-) *N*-methamphetamine, which is available over the counter as a nasal decongestant [3], and (+) S-methamphetamine, a DEA Schedule I controlled substance, *N*-ethyl amphetamine, also a DEA Schedule I controlled substance, was sold as the racemic material [2]. Substances with this classification have no current accepted medical use [1]. *N*-ethyl amphetamine is metabolized to amphetamine or excreted unchanged. Of the two enantiomers, the (+) enantiomer is metabolized to a greater extent [4]. The racemate has been available commercially in small quantities for analytical purposes. 

**Figure 1:**
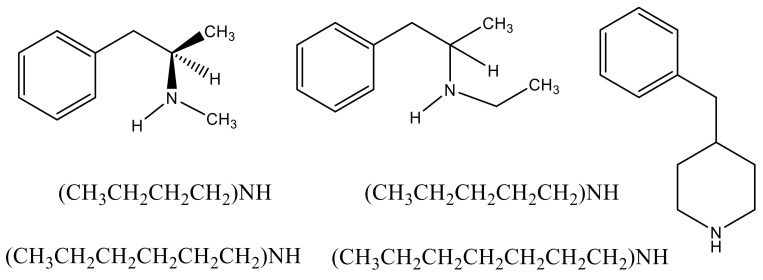
Left to right: (*dl*) S (+)-methamphetamine, (*dl*)-*N*-ethylamphetamine, 4-benzylpiperidine; *N*,*N*-dibutylamine, *N*,*N*-dipentylamine; *N*,*N*-dihexylamine, *N*,*N*-diheptylamine.

Despite the use and abuse of this drug, very little information is available about its properties, presumably because of the restrictions regarding its accessibility. Our laboratory has been investigating the use of a technique referred to a correlation gas chromatography to evaluate both vaporization enthalpies and vapor pressures of materials that are either difficult to access, or beyond the current capabilities of most conventional techniques used for these purposes. This includes substances that are present in complex mixtures [5,6], are either quite non-volatile [7], or as in this case, available only in very small quantities [8]. This work describes our results for (±) *N*-ethylamphetamine whose structure is given in Figure 1 along with the materials used as standards.

## 2. Experimental Methods

The origin of the materials used, their CAS numbers and their composition in mass fraction are provided in Table 1. Two of the materials were also analyzed by gas chromatography. Analysis of the remaining compounds were provided by their suppliers. The solvent used was methanol. A methanolic solution of the standards were prepared by dissolving a drop of each into a small vial containing 1 mL of methanol and adding several drops of this solution to approximately a 0.5 mL of the methanolic solution of (*dl*)-*N*-ethylamphetamine until comparable amounts of standards and target were achieved; composition was monitored by gas chromatography. Each compound in the mixture was identified independently by its retention time. 

### 2.1. Analytical Methods

An HP 5890 gas chromatograph running HP Chemstation and equipped with a 15 m Supelco SPB-5 capillary column (0.32 mm, 1.0 μm film thickness) at a split ratio of approximately 80/1 was used for the analysis and the measurements. Helium was used as the carrier gas at a head pressure of approximately 150 kPa. Column temperature was controlled by the instrument to (±0.1) K as monitored by a high temperature probe connected to a Go Link^TM^ interface. At the temperatures of the experiments, methanol is not retained by the column as evidenced by a slight increase in its retention time with temperature. This increase is due to the increase in viscosity of the carrier gas with temperature and serves as a measure of the flow through the column. Isothermal chromatograms were recorded at *T* = 5 K intervals over a 30 K temperature range. All retention times recorded are provided in the Supplementary Materials, Table S1–S3.

### 2.2. Thermochemical Methods: Vaporization Enthalpies

A basic premise in correlation gas chromatography, is that the time an analyte spends on the column, its resident time, *t*_r_, is inversely proportional to its vapor pressure *p*, off the column. If 1/*t*_r_ is proportional to to *p*, then ln (1/*t*_r_) can be related to K/*T*, by the Clausius Clapeyron relationship. Residence times can be evaluated by the difference in retention times of the analyte and a non-retained reference, in this case, methanol. Plots of ln (*t*_o_/*t*_r_) vs K/*T*, where *t*_o_ is the reference time, 60 s, results in a linear relationship, the slope of which is related to an enthalpy when multiplied by the gas constant, -*R* (8.314 J·mol^−1^·K^−1^). This enthalpy, ${∆}_{trn}^{g}H_{m}\left(T_{m}\right),$ is related to the vaporization enthalpy by Equation (1), where ${∆}_{l}^{g}H_{m}\left(T_{m}\right)$ refers to the vaporization enthalpy and $∆H_{intr}\left(T_{m}\right)$ is a measure of the interaction of each analyte with the column [9]. The term, ${∆}_{trn}^{g}H_{m}\left(T_{m}\right)$, is referred to as the enthalpy of transfer of the analyte from the stationary phase of the column to the gas phase. The non-retained reference is identified by its temperature dependence of *t*_r_, which contrary to retained substances, actually increases slightly with temperature due to the slight increase in viscosity of the carrier gas.

(1) $${∆}_{trn}^{g}H_{m}\left(T_{m}\right)={∆}_{l}^{g}H_{m}\left(T_{m}\right)+∆H_{\mathrm{intr}}\left(T_{m}\right)$$

When materials similar to the target (s) with known vaporization enthalpies are included in the chromatography, a plot of ${∆}_{l}^{g}H_{m}\left(298.15\;K\right)$ vs. ${∆}_{trn}^{g}H_{m}\left(T_{m}\right)$ is linear. The slope and intercept of the line together with the value of ${∆}_{trn}^{g}H_{m}\left(T_{m}\right)$ of the target can be used to evaluate ${∆}_{l}^{g}H_{m}\left(298.15\;K\right)$ of the target. The structure of the hydrocarbon portion of the standards may vary as long as the number and type of functional groups present in the targets are matched. Best results are obtained with homologous series [10,11].

### 2.3. Thermochemical Methods: Vapor Pressures

The relationship between *t*_o_/*t*_r_ and the vapor pressure of the material off the column also suggests a possible relationship between *t*_o_/*t*_r_ and *p*, the vapor pressure of the pure material, provided appropriate standards are chosen functionally related to the target (s). We have found that a plot of ln (*p*/*p*^o^) against ln (*t*_o_/*t*_r_), where *p*^o^ is a reference pressure, in this work 101,325 Pa, a linear relationship is obtained which frequently persists over a range of temperatures. The parameters of this relationship together with the values of ln (*t*_o_/*t*_r_) of the target (s) can afford the vapor pressure (s) of the targets in a fashion similar to what has been described above. As with vaporization enthalpy, the quality of the results are depended on the similarity between standards and targets and the quality of the properties that are being correlated.

### 2.4. Uncertainties

All uncertainties reported in this work refer to one standard deviation unless noted otherwise [12]. Linear and non-linear least squares were performed by Excel and Sigma Plot 14, respectively. All regression coefficients (r^2^) not described below exceeded 0.99. Uncertainties obtain from logarithmic relationships are reported as an average value. The uncertainties reported are generally a measure of the quality of the correlation; actual uncertainties in the values reported may vary. Evaluation of the uncertainty in the reported boiling temperature is described below. Whenever possible, uncertainties in combined results are evaluated as the square root of the sum of the squares of the uncertainty of each contributing term.

### 2.5. Estimation of Vaporization Enthalpy

An estimate of the potential vaporization enthalpy is quite helpful in identifying either unanticipated interactions [13] or unreasonable experimental results. A very simple yet quite useful equation for evaluating relatively simple molecules is given by Equation (2) [14]. The terms *n*_C_ in Equation (2) defines the total number of carbon atoms and and *n*_Q_, the number of quaternary sp^3^ hybridized carbon atoms, respectively. The b term identifies the contribution of the functional group, for a secondary amine, b = 8.9 kJ·mol^−1^ and the C term for compounds containing a single functional group corrects for each carbon branch off an sp^3^ hybridized carbon present, C = −2.0 kJ·mol^−1^· branch^−1^. For N-ethylamphetamine, *n*_c_ = 11, *n*_Q_ = 0, and there are no carbon branches on an sp^3^ hybridized carbon atom resulting in a vaporization enthalpy at *T* = 298.15 K of (63.5 ± 3.2) kJ·mol^−1^. The uncertainty associated with the estimation is general 5% of the value estimated.

(2) $${∆}_{l}^{g}H_{m}\left(298.15\;K\right)/\mathrm{kJ}\cdot {\mathrm{mol}}^{-1}=4.69\left(n_{c}-n_{Q}\right)+1.3n_{Q}+b+3.0+C$$

### 2.6. Vaporization Enthalpy: Temperature Adjustments

Vaporization enthalpies of the standards are summarized in Table 2. Not all are available at *T* = 298.15 K. Temperature adjustments for those reported at different temperatures have been adjusted to *T* = 298.15 K according to Equation (3). Heat capacities for these adjustments were evaluated by group additivity using the protocol summarized in Reference [15]. For compounds with more than one vaporization enthalpy cited, an average value was used in subsequent correlations.

(3) $${∆}_{l}^{g}H_{m}\left(298.15\;K\right)/\mathrm{kJ}\cdot {\mathrm{mol}}^{-1}={∆}_{l}^{g}H_{m}\left(T_{m}\right)/\mathrm{kJ}\cdot {\mathrm{mol}}^{-1}+[(10.58+0.26\cdot C_{p}(l,\;298\;K)/(J\cdot {\mathrm{mol}}^{-1}\cdot K^{-1}))(T_{m}/K-298.15\;K)]/1000$$

### 2.7. Vapor Pressures

Vapor pressures of the *N*,*N*-dialkylamines are all available in the form of the Antoine equation, Equation (4). Table 3 lists the constants of this equation and the temperature range at which these constants are applicable. The vapor pressures of 4-benzylpiperidine, listed at the bottom of the table, are expressed in the form of a third order polynomial, Equation (4). The constants of this polynomial are also provided. As indicated in the table, only one set of Antoine constants are applicable at ambient temperatures. We have previously observed however, that the vapor pressures generated by these constants, correlated quite well as a function of temperature [8]. With the exception of 4-benzylpiperidine, all amines are members of a homologous series. Since the vapor pressures of the first member of the series is valid over a broad range of temperatures, the quality of the correlations as a function of temperature suggests that vapor pressures resulting from extrapolations of the remaining members are relatively reliable.

Antoine Equation ln(*p*/kPa) = A_A_ − B_A_/(*T*/K + C_A_) (4)

ln(*p*/*p*^o^) = A_T_ + B_T_(*T*/K) + C_T_(*T*/K)^2^ + D_T_(*T*/K)^3^; *p*^o^ = 101,325 Pa (5)

## 3. Results 

### 3.1. Vaporization Enthalpies 

Plots of ln (*t*_o_/*t*_r_) vs. K/*T* of each analyte at 5 K intervals over a 30 K temperature range were linear, characterized by correlation coefficients r^2^ > 0.999. Enthalpies of transfer were calculated as the product of the absolute value of the slope of the line and the gas constant. Vaporization enthalpies of the standards plotted against the enthalpies of transfer were also linear. Vaporization enthalpy of the target was calculated as the sum of the product of the slope of the line and the enthalpy of transfer and the intercept, Equation (6). Table 4 summarizes the results of one of two duplicate runs. Results of the second run are reported in the SM. Results for both runs are summarized in Table 5 and are discussed below. 

### 3.2. Vapor Pressures 

With the exception on 4-benzypiperidine, the vapor pressures of all other standards are available in the form of the Antoine equation. Vapor pressures calculated using Equation (4) were first converted to Pa and then correlated in the form ln( *p*/*p*^o^) where *p*^o^ refers to 101,325 Pa. Runs 1 and 2 were both performed under similar experimental conditions. Values of *t*_o_/*t*_r_ of each respective analyte, calculated from the slopes and intercepts of Tables S2 and S4 (see Supplementary Materials) were averaged, and then ln (*p*/*p*^o^) of the standards were correlated against their respective ln (*t*_o_/*t*_r_)_avg_ values at *T* = (298.15, 310) K and at 10 K increments up to 450 K; the correlations also included 4-benzypiperidine. The resulting correlation equation at each temperature together with ln (*t*_o_/*t*_r_)_avg_ of N-ethylamphetamine was used to evaluate its corresponding vapor pressure. All correlation coefficients (r^2^) exceeded 0.9968 over this temperature range. Table 6 illustrates the correlation performed at *T* = 298.15 K.

The resulting values of ln (*p*/*p*^o^) as a function of temperature were then fit to a second order polynomial, Equation (8). The resulting constants of Equation (8) are provided in Table 7.

ln(*p*/*p*^o^) = A_S_ + B_S_(*T/K*) + C_S_(*T/K*)^2^; *p*^o^ = 101,325 Pa (8)

### 3.3. Estimation of Boiling Temperatures and Their Uncertainties

Boiling temperatures were evaluated by extrapolating Equation (8) until ln (*p*/*p*^o^) = 0. Uncertainties in boiling temperature were evaluated by combining each vapor pressure evaluated from *T* = (298.15 to 450) K with its respective uncertainty, *p*_u_ (1σ), and fitting the results in the form ln ((*p* + *p*_u_)/*p*^o^) to a second polynomial. Solving for temperature at which this term equaled zero, the uncertainty in boiling temperature was evaluated as the difference between the two temperatures evaluated. 

## 4. Discussion

### 4.1. Vaporization Enthalpy

The results of two correlations used evaluate the vaporization enthalpy of N-ethylamphetamine are summarized in Table 5. The value of 62.4 ± 4.4 kJ·mol^−1^ is in close agreement with the value (63.5 ± 3.8) kJ·mol^−1^, estimated using Equation (2). For comparison, the vaporization enthalpy of *S* (+)-methamphetamine has been reported as (58.7 ± 4.3) kJ·mol^−1^. The difference of (3.7 ± 5.8) kJ·mol^−1^ is within the typical increment in vaporization enthalpy observed for the insertion of a methylene group. Values as small as (0.9 ± 1.0) kJ·mol^−1^ have been observed between liquid ethyl and methyl *p*-aminobenzoate [23,24] and as large as (5.004 ± 0.008) kJ·mol^−1^/CH_2_ group in vaporization enthalpy going from pentane to octatriacontane [25].

### 4.2. Vapor pressure

**Figure 2:**
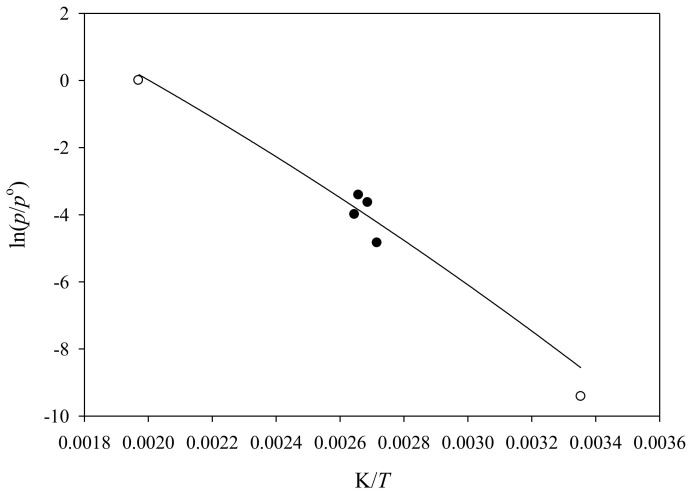
Line: a plot of ln (*p*/*p*^o^) vs. K/*T* of N-ethylamphetamine from *T* = (298.15 to 500) K; ●: experimental boiling temperatures at reduced pressures (most volatile to least volatile [26,27,28,29]; o: estimated values [21].

Vapor pressure results for N-ethylamphetamine are reported in Table 6 and Table 7. A vapor pressure of (19 ± 11) Pa compares to a value of 38 Pa evaluated for S (+)-methamphetamine at *T* = 298.15 K. Similarly, estimated boiling temperatures at *p* = 101,325 Pa of *T* = (490 [8], 489 [21]) K for S (+)-methamphetamine compare to a corresponding estimated temperature of *T* = (499 ± 3 (this work), 508 [21]) K. Additionally, several experimental boiling temperatures at reduced pressures have been reported for *N*-ethylamphetamine. Figure 2 provides a qualitative comparison of these values to the values evaluated in this work. The solid circles represent experimental boiling temperatures at reduced pressures while the remainder are estimated values. A numerical comparison of the vapor pressures of *N*-ethylamphetamine to S (+)-methamphetamine over the temperature range *T* = 298,15 to *T*_B._ the normal boiling temperature, is provided as Table S5 (Supplementary Materials).

## 5. Summary

Table 8 summarizes the vaporization enthalpy, vapor pressure, the constants of Equation (8) that can be used to predict the vapor pressures of N-ethylamphetamine as a function of temperature and the predicted boiling temperature at *p*^o^ = 101,325 Pa.

## Figures and Tables

**Table 1:** Origin of the standards and *d*-methamphetamine and their analysis.

Compound	CAS Registry No.	Supplier	Mass Fraction Supplier	GC
Methanol	67-56-1	Fisher Scientific	0.998	
*N*,*N*-Dibutylamine	111-92-2	Aldrich	0.99	
(*dl*)-*N*-Ethylamphetamine	457-87-4	Millipore Sigma	reference standard	>0.99 ^1^
*N*,*N*-Dipentylamine	2050-92-2	TCI	>0.97	
4-Benzylpiperidine	31252-42-3	Aldrich	0.99	
*N*,*N*-Dihexylamine	143-16-8	TCI		>0.99
*N*,*N*-Diheptylamine	2470-68-0	TCI	>0.98	

^1^ Available in a sealed ampule in methanol, (1 mg/mL).

**Table 2:** Temperature adjustments of literature vaporization enthalpies.

	${∆}_{l}^{g}H_{m}\left(T_{m}\right)\;\mathrm{kJ}\cdot {\mathrm{mol}}^{-1}$	*T*_m_/K	*C*_p_(l, 298 K) ^1^ J·mol^−1^·K^−1^	Δ*C*_p_Δ*T* ^2^ kJ·mol^−1^	${∆}_{l}^{g}H_{m}\left(298\;K\right)\;\mathrm{kJ}\cdot \mathrm{mol}{-}^{1}$	Ref.
N,N-Dibutylamine	50.8 ± 4.1	298.15	308.2		50.8 ± 4.1	[8]
	46.0	343.2	308.2	4.1 ± 1.0	50.1 ± 1.0	[16]
	44.75	358.2	308.2	5.4 ± 1.0	50.2 ± 1.0	[16]
	49.4 ± 0.1	298.15	308.2		49.4 ± 0.1	[17]
Average					50.1 ± 0.6 ^3^	
N,N-Dipentylamine	61.2 ± 2.6	298.15	372		61.2 ± 2.6	[8]
	51.2	394	372	10.3 ± 1.6	61.4 ± 1.6	[18]
Average					61.3 ± 2.1 ^3^	
N,N-Dihexylamine	70.8 ± 4.7	298.15	435.8		70.8 ± 4.7	[8]
	55.1	423	435.8	15.5 ± 2.1	70.5 ± 2.1	[17,18]
Average					70.7 ± 3.4 ^3^	
4-Benzylpiperidine	74.2 ± 1.0	298			74.2 ± 1.0	[19]
N,N-Diheptylamine	60.0	450	499.6	21.3 ± 2.6	81.3 ± 2.6	[18]
	81.2 ± 7.1	298.15	499.6		81.2 ± 7.1	[8]
Average					81.3 ± 4.9 ^3^	

^1^ Heat capacities evaluated by group additivity [14]; no experimental heat capacities were located in Reference [20]. ^2^ Temperature adjustments were evaluated using the following equation for liquids [15]. ^3^ One standard deviation (±1 σ) associated with the values cited.

**Table 3:** Constants for the Antoine equation (and of a third order polynomial; *p*^o^ = 101,325 Pa).

Antoine Equation (4)	A_A_	B_A_	−C_A_	*T*_range_/K	*p*_(l, 298 K)_/Pa	Ref.
*N*,*N*-Dibutylamine	14.6511	3687.84	65.37	286–371	304	[16]
*N*,*N*-Dipentylamine	14.7935	4105.74	72.15	379–527	34	[18]
*N*,*N*-Dihexylamine	15.1013	4635.56	69.15	408–569	5.8	[18]
*N*,*N*-Diheptylamine	14.8948	4716.85	86.15	435–605	0.64	[18]
**3rd Order Polynomial Equation (5)**	**A_T_**	**B_T_**	C_T_ × 10^−4^	**D_T_ × 10^−6^**	***p*_(l, 298 K)_/Pa**	
4-Benzylpiperidine	6.74	−1642.3	−130.851	62.508	1.5	[8]
(S) (+)-Methamphetamine	7.592	–2119.6	–84.929	31.824	39	[8]

**Table 4:** Correlation between ${∆}_{l}^{g}H_{m}\left(298.15\;K\right)$ and ${∆}_{\mathrm{trn}}^{g}H_{m}$ (408 K) of the standards ^1^.

Run 1	−Slope *T*/K	Intercept	${∆}_{trn}^{g}H_{m}(408\;K)$ kJ·mol^−1^	${∆}_{l}^{g}H_{m}\left(298.15\;K\right)/\mathrm{kJ}\cdot {\mathrm{mol}}^{-1}$	
				(lit) ^2^	(calc)
*N*,*N*-Di-n-butylamine	4197.2 ± 20	10.984 ± 0.05	34.89 ± 0.16	50.1 ± 0.6	50.4 ± 3.4
*N*,*N*-Di-n-pentylamine	5121.3 ± 23	12.181 ± 0.06	42.58 ± 0.19	61.3 ± 2.1	61.0 ± 3.7
*N*-Ethylamphetamine	5238.1 ± 24	11.973 ± 0.06	43.55 ± 0.20		62.4 ± 3.8
*N*,*N*-Di-n-hexylamine	6037.1 ± 24	13.374 ± 0.06	50.19 ± 0.20	70.7 ± 3.4	71.5 ± 4.0
4-Benzylpiperidine	6149.2 ± 27	12.810 ± 0.07	51.12 ± 0.22	74.2 ± 1.0	72.8 ± 4.1
*N*,*N*-Di-n-heptylamine	6937.5 ± 36	14.546 ± 0.09	57.68 ± 0.30	81.3 ± 4.9	81.8 ± 4.3

${∆}_{l}^{g}H_{m}\left(298.15\;K\right)$/kJ·mol^−1^ = (1.38 ± 0.06) ${∆}_{trn}^{g}H_{m}$(408 K) + (2.3 ± 2.8)				r^2^ = 0.9947 (6)

^1^ Uncertainties represent one standard deviation. ^2^ References are reported in Table 2.

**Table 5:** Summary: Vaporization Enthalpy (kJ·mol^−1^) at *T* = 298.15 K ^1^.

	Run 1	Run 2	Average	Lit. ^2^	Estimate
*N*,*N*-Di-n-butylamine	50.4 ± 3.4	50.2 ± 4.5	50.3 ± 4.0	50.1 ± 0.6 ^3^	49.4 ± 2.4
*N*,*N*-Di-n-pentylamine	61.0 ± 3.7	60.9 ± 4.9	61.0 ± 4.3	61.3 ± 2.1 ^4^	58.8 ± 2.9
*N*-Ethylamphetamine	62.4 ± 3.8	62.3 ± 4.9	62.4 ± 4.4		63.5 ± 3.2
*N*,*N*-Di-n-hexylamine	71.5 ± 4.0	71.8 ± 5.3	71.7 ± 4.7	70.7 ± 3.4 ^4^	68.2 ± 3.4
4-Benzylpiperidine	72.8 ± 4.1	73.3 ± 5.4	73.1 ± 4.8	74.2 ± 1.0 ^5^	68.2 ± 3.4
*N*,*N*-Di-n-heptylamine	81.8 ± 4.3	82.5 ± 5.8	82.2 ± 5.1	81.3 ± 4.9 ^4^	77.6 ± 3.9

^1^ Uncertainties represent one standard deviation. ^2^ See Table 2 for details. ^3^ Average from References [8,16,17]. ^4^ Average from References [8,18]. ^5^ Reference [19].

**Table 6:** Correlations of ln (*p*/*p*^o^) vs ln (*t*_o_/*t*_r_) at *T* = 298.15 K.

	ln(*t_o_*/*t_r_*)	ln(*p*/*p*^o^)	ln(*p*/*p*^o^)	*p*/Pa	*p*/Pa/Lit	Lit.
*N*,*N*-Di-n-butylamine	−7.739	−5.810	−5.83 ± 0.49	300 ± 120	304	[16]
*N*,*N*-Di-n-pentylamine	−9.634	−7.992	−7.90 ± 0.53	37 ± 20	34	[18]
*N*-Ethylamphetamine	−10.235		−8.56 ± 0.54	19 ± 11	8.2 ^1^	[21]
*N*,*N*-Di-n-hexylamine	−11.517	−9.760	−9.97 ± 0.58	4.7 ± 2	5.8	[18]
4-Benzylpiperidine	−12.427	−11.130	−10.97 ± 0.60	1.8 ± 1	1.5	[8]
*N*,*N*-Di-n-heptylamine	−13.369	−11.973	−12.00 ± 0.62	0.62 ± 0.4	0.64	[18]

$\ln\left(p/p^{o}\right)=\left(1.096\pm 0.036\right)\cdot \ln\left(t_{0}/t_{r}\right)+\left(2.616\pm 0.40\right)$					r^2^ = 0.9968 (7)	

^1^ Estimate.

**Table 7:** Coefficients of the second order polynomial, Equation (8); *p*^o^ = 101,325 Pa.

	A_S_	B_s_	C_s_	*T*_B_/K	*T*_B_^Lit^/K
*N*,*N*-Di-n-butylamine	9.483 ± 0.02	−3128.7 ± 12	−427980 ± 2190	434 ± 5	432.8 ^1^
*N*,*N*-Di-n-pentylamine	9.395 ± 0.03	−3271.1 ± 19	−562,260 ± 3409	474 ± 3	475.2 ^1^
*N*-Ethylamphetamine	8.347 ± 0.01	−2872.4 ± 3.7	−646650 ± 665	499 ± 3	508 ^2^
*N*,*N*-Di-n-hexylamine	9.483 ± 0.04	−3128.7 ± 27	−427,980 ± 4919	512 ± 2	506–516 ^1^
4-Benzylpiperidine	7.429 ± 0.01	−2679.1 ± 10	−836,480 ± 1734	561 ± 1	552 ^3^
*N*,*N*-Di-n-heptylamine	9.309 ± 0.05	−3582.1 ± 36	−825,650 ± 6424	547 ± 1	544.2 ^1^

^1^ Reference [8]. ^2^ Estimate, reference [21]. ^3^ Reference [22].

**Table 8:** A Summary of the Vaporization Enthalpy, Vapor Pressure at 298.15 K, Coefficients of Equation (8) and Predicted Boiling temperature at po =101,325 Pa of N-Ethylamphetamine.

${∆}_{l}^{g}H_{m}\left(298\;K\right)/\mathrm{kJ}\cdot {\mathrm{mol}}^{-1}$	*p*/Pa	*A_S_*	*B_s_*	*C_s_*	*T*_B_/K
62.4 ± 4.4	19 ± 11	8.347 ± 0.01	–2,872.4 ± 3.7	–646650 ± 665	499 ± 3

## Supplementary Materials

The following are available online. Tables S1 and S3: Retention times of *N*-Ethylamphetamine and Standards; Tables S2 and S4: Correlations of ${∆}_{trn}^{g}H_{m}\left(T_{m}\;K\right)$ with ${∆}_{l}^{g}H_{m}\left(298\;K\right)$ of the Standards; Table S5: A Comparison of the Vapor Pressures of (S) (+)-Methamphetamine to *N*-Ethylamphetamine as a Function of Temperature.
