# Supplementary material for: The Vaporization Enthalpy and Vapor Pressure of (±) N-Ethyl Amphetamine by Correlation Gas Chromatography
Source: Molecules. 2021 Jun 22;26(13):3809. doi: 10.3390/molecules26133809 (PMC8270283; doi:10.3390/molecules26133809)
Supplement: Supplementary file 1 [file molecules-26-03809-s001.zip › molecules-1247532-supplementary.pdf]

molecules

Supporting Materials

The Vaporization Enthalpy and Vapor Pressure of (±)-N-Ethylamphetamine at *T*/K = 298.15 by Correlation Gas Chromatography

James Chickos

Department of Chemistry and Biochemistry, University of Missouri-St. Louis, St. Louis MO 63121

Table S1. A: Retention times of N-ethylamphetamine and compounds used as standards.

Run 1	*T*/K 392.9	397.9	403	408.1	413.1	418.1	423.2
Methanol	0.517	0.525	0.529	0.524	0.062	0.538	0.542
N,N-Di-n-butylamine	1.261	1.172	1.095	1.020	0.500	0.928	0.887
N,N-Di-n-pentylamine	2.879	2.518	2.223	1.967	1.300	1.611	1.470
N-Ethylamphetamine	4.434	3.814	3.317	2.891	2.083	2.285	2.048
N,N-Di-n-hexylamine	7.893	6.562	5.524	4.656	3.507	3.453	2.992
4-Benzylpiperidine	17.772	14.578	12.128	10.094	7.999	7.239	6.157
N,N-Di-n-heptylamine	23.179	18.485	14.970	12.139	9.482	8.321	6.930

Table S1. B: Correlation between $\Delta_l^g H_m$(298.15 K) and ΔH_{trn}(408K) of the standards[1]

Run 1	-slope *T*/K	intercept	ΔH_{trn}(408K) kJ·mol^{-1}	$\Delta_l^g H_m$(298.15 K)/kJ·mol^{-1} (lit)[2]	(calc)
N,N-Di-n-butylamine	4197.2±20	10.984±0.05	34.89±0.16	50.1±0.6	50.4±3.4
N,N-Di-n-pentylamine	5121.3±23	12.181±0.06	42.58±0.19	61.3±2.1	61.0±3.7
N-Ethylamphetamine	5238.1±24	11.973±0.06	43.55±0.20		62.4±3.8
N,N-Di-n-hexylamine	6037.1±24	13.374±0.06	50.19±0.20	70.7±3.4	71.5±4.0
4-Benzylpiperidine	6149.2±27	12.810±0.07	51.12±0.22	74.2±1.0	72.8±4.1
N,N-Di-n-heptylamine	6937.5±36	14.546±0.09	57.68±0.30	81.3±4.9	81.8±4.3

$\Delta_l^g H_m(298.15\ K)$/kJ·mol^{-1} = (1.38±0.06)ΔH_{trn}(408 K) + (2.3±2.8) $r^2 = 0.9947$ (S1)

[1] All uncertainties represent one standard deviation.

[2] Vaporization enthalpies are average literature values; individual values and references are reported in Table 2.

Table S2. A: Retention times of N-ethylamphetamine and compounds used as standards.

Run 2	392.9	398	403.1	408.2 t/min	413.2	418.2	423.2
Methanol	0.541	0.536	0.551	0.545	0.549	0.557	0.559
N,N-Di-n-butylamine	1.315	1.211	1.142	1.049	1.006	0.960	0.919
N,N-Di-n-pentylamine	2.998	2.613	2.318	2.035	1.842	1.670	1.527
N-Ethylamphetamine	4.614	3.966	3.459	2.996	2.663	2.371	2.130
N,N-Di-n-hexylamine	18.524	15.237	12.648	10.488	8.871	7.495	6.405
4-Benzylpiperidine	24.132	19.318	15.625	12.647	10.437	8.624	7.212
N,N-Di-n-heptylamine	24.132	19.318	15.625	12.647	10.437	8.624	7.212

Table S2. B: Correlation between $\Delta_l^g H_m$(298.15 K) and ΔH_{trn}(408K) of the standards [1]

Run 2	-slope *T*/K	intercept	ΔH_{trn}(408K) kJ·mol⁻¹	ΔH_{vap}(298 K) kJ·mol⁻¹ (lit)	ΔH_{vap}(298 K) kJ·mol⁻¹ (calc)
N,N-Di-n-butylamine	4214.4±74	10.987±0.18	35.04±0.62	50.1±0.6	50.2±4.5
N,N-Di-n-pentylamine	5120.2±31	12.137±0.07	42.57±0.25	61.3±2.1	60.9±4.9
N-Ethylamphetamine	5236.5±22	11.927±0.05	43.53±0.18		62.3±4.9
N,N-Di-n-hexylamine	6041.9±25	13.342±0.06	50.23±0.21	70.7±3.4	71.8±5.3
4-Benzylpiperidine	6171.3±13	12.844±0.03	51.31±0.11	74.2±1.0	73.3±5.4
N,N-Di-n-heptylamine	6950.4±12	14.533±0.03	57.78±0.10	81.3±4.9	82.5±5.8

ΔH_{vap}(298.15 K)/kJ·mol^{-1} = (1.42±0.08)ΔH_{trn}(408 K) + (0.46±3.56) r^2 = 0.9939 S2)

[1] All uncertainties represent one standard deviation.

[2] Vaporization enthalpies are average literature values; individual values and references are reported in Table 2.

Table S3. A comparison of the vapor pressures of (S) (+)-methamphetamine to N-ethylamphetamine as a function of temperature [1].

(S) (+)-Methamphetamine *T*/K		N-Ethylamphetamine *p*/Pa[2]	(S) (+)-Methamphetamine *T*/K		N-Ethylamphetamine *p*/Pa[2]
298.15	*39*	19±11 (8.2) [S1]	390	*5600*	3900±870
310	*91*	48±24	400	*8200*	**5700**±1200
320	*180*	98±45	410	*12000*	8300±1500
330	*320*	190±77	420	*16000*	12000±1900
340	*570*	340±130	430	*22000*	16000±2400
350	*960*	590±200	440	*29000*	22000±2900
360	*1600*	1000±310	450	*39000*	30000±3400
368.2		1500±410 (800)[S2]	460	*50000*	39000
370	*2500*	1600±450	470	*64000*	51000
372.2		1800±480 (2700) [S3]	480	*81000*	65000
376.2		2100±560 (3300) [S4]	490	*101300*	82000
378.2		2300±600 (1900) [S5]	500		101300
380	*3800*	2500±630	507.7		120000 (101325) [S1]

[1] N-Methylamphetamine: vapor pressures evaluated using Eq. (S1);

$\ln(p/p^o) = 7.592 - 2119.6(T/K) – 849,290(T/K)^2 - 31,824,000(T/K)^3$; p^o = 101,325 Pa (S3)

N-ethylamphetamine: vapor pressure from T = (298.15 to 450) K evaluated by correlation;

from T = (460 to 500) K: $\ln(p/p^o) = (8.347±0.01) – (2,872.4±3.7)(T/K) – (646,650±665)(T/K)^2$ (S4)

[2] Uncertainties are a measure of the quality of the correlation; actual uncertainties may differ._

References

[S1] Estimate, EPI Suite version 4.11 (Estimation Programs Interface), The EPI Suite can be downloaded at http://www.epa.gov/oppt/exposure/pubs/episuitedl.htm; accessed 4/08/21.

[S2] Chiavarelli, S.; Marini-Bettolo, G. B. Synthetic sympatholytic substances in the ergotamine series. II. Derivatives of benzylamine, phenethylamine, and α-methylphenethylamines with amine and amide functions. Gazzetta Chimica Italiana **1951**, *81*, 89-97.

[S3] Shiho, D.; Kanayama, K.; A new process of alkylation of amines. II. Nippon Kagaku Kaishi 1944, 65, 237-239.

[S4] Temmler, T. H.; Keil, F.; Dobke, W. β-Aralkylamines. DE 767263 **1952.**

[S5] Leonard, N. J.; Adamcik, J. A.; Djerassi, C.; Halpern, O. Cyclic amino acyloins and amino ketones. X. Trans-annular nitrogen-carbonyl interaction in cyclic aminoketones and optical rotatory dispersión. *J. Am. Chem. Soc*. **1958**, *80*, 4858-4862.
